# Development of Porous Silicon(Si) Anode Through Magnesiothermic Reduction of Mesoporous Silica(SiO_2_) Aerogel for All-Solid-State Lithium-Ion Batteries

**DOI:** 10.3390/gels11040304

**Published:** 2025-04-21

**Authors:** Pratik S. Kapadnis, Kangsanin Kim, Kisun Nam, Yongseon Kim, Hyung-Ho Park, Haejin Hwang

**Affiliations:** 1 Department of Materials Science and Engineering, Inha University, Incheon 22212, Republic of Korea; pratiknanoworld@gmail.com (P.S.K.); kks6625@gmail.com (K.K.); skarltjs2@naver.com (K.N.); ys.kim@inha.ac.kr (Y.K.); 2 Department of Materials Science and Engineering, Yonsei University, Seoul 03722, Republic of Korea; hhpark@yonsei.ac.kr

**Keywords:** hydrophobic/hydrophilic SiO_2_, porous Si, magnesiothermic reduction, anode, all-solid-state lithium-ion battery

## Abstract

All-solid-state lithium-ion batteries (ASSLBs) are attractive energy storage devices because of their excellent gravimetric and volumetric capacity and ability to supply high power rates. Porous silicon (Si) is a promising material for an anode in lithium-ion batteries due to its high capacity and low discharge potential. However, Si anodes cause significant problems due to strong volume growth during the lithiation and delithiation processes, which results in rapid capacity fading and poor cycle stability. To overcome this problem, we developed mesoporous silica (SiO_2_) aerogels into porous silicon (Si) anodes using a magnesiothermic reduction (MTR) process. By effectively preserving the porous structure, this approach enables the material to endure volume fluctuations while maintaining its structural integrity during cycling. In our study, we demonstrated a feasible approach to fabricate the porous silicon (Si) from hydrophobic and hydrophilic silica (SiO_2_) aerogel and magnesium powder (Mg) through the MTR process at 600~900 °C. The sample obtained after the reduction process was treated with hydrochloric acid (HCl) to remove byproducts. As prepared, Si was characterized using various techniques, including XRD, XRF, FT-IR, XPS, SEM, and BET, which confirmed the successful production, chemical purity, and structural retention of Si. Furthermore, the coin cell was fabricated using Si as an anode, and the electrochemical performance was analyzed. The charge/discharge cycling tests at 1 C and 0.02~2 V (vs. the Li condition) revealed the effects of silicon content, wettability, and interfacial compatibility on electrode performance. Conversely, for better understanding, a long-term cycling test was conducted at 1 C rate, 0–1.5 V (vs. Li) to evaluate capacity retention. Our findings highlight the potential application of silicon (Si) aerogels produced from silica (SiO_2_) aerogels by magnesiothermic reduction to improve lithium-ion battery performance.

## 1. Introduction

Lithium-ion batteries are attractive energy storage devices because of their excellent gravimetric and volumetric capacity and ability to supply high power rates [1]. The increasing demand for high-performance energy storage devices led to substantial research into improved lithium-ion battery (LIB) materials [2]. Lithium-ion batteries (LIBs) have become the recommended chemistry because of their high energy and power density [3]. High energy, power density, enhanced safety, and low cost are all highly desired outcomes of developing lithium-based rechargeable batteries [4,5]. The anode material of currently commercialized lithium secondary batteries is graphite, which has high electronic conductivity, low cost, and a structural arrangement favorable for inserting lithium ions [6]. However, graphite has low energy density and capacity, which limits the battery′s overall capacity and has the disadvantage of forming lithium dendrites [7].

Silicon (Si) is a promising material to use as an anode in lithium-ion batteries due to its high capacity (3579~4200 mAh/g^−1^) and low discharge potential. Si reacted with lithium-ions expands in volume during the alloying process, which results in the pulverization and exfoliation of Si particles [8,9,10]. However, Si anodes have significant challenges, especially due to high volume expansion (>300%) during the lithiation and delithiation processes, leading to rapid capacity fading and poor cycle stability [11]. The pulverization of Si particles leads to continuous interaction with the electrolyte, which subsequently creates a formation reaction of a series of solid electrolyte interphases. This reaction results in low capacity or irreversible capacity relative to the theoretical capacity, as well as low initial Coulombic efficiency (ICE) [12]. Further studies demonstrated that one approach is to synthesize the Si material with different nanostructures, such as nanoparticles [13,14], nanowires [15], nanotubes [16], nanosheets [17], and porous nanostructures [18,19] which are capable of significant volume fluctuation.

Porous morphologies can potentially address the challenges of volumetric expansion and slow lithium diffusion. The porous structure can reduce volume expansion and mechanical stress by generating a space for volume expansion, resulting in superior cycle performance [20]. Additionally, Si nanoparticles have a large surface area compared to their weight, providing more lithium-ion reactions and increasing the contact area with the electrolyte, reducing lithium-ion diffusion ranges and enhancing usable capacity.

On the other hand, spherical silica aerogel (SiO_2_) is a super porous material with high porosity, low density, low thermal conductivity [21], and a large specific surface area with a 3D network structure [22,23].

Porous silicon (Si) can be produced by reducing mesoporous silica aerogel (SiO_2_) while maintaining its structural changes, which may reduce the volume expansion of Si that occurs while filling the internal and surface pore [24]. Promising metal-reducing agents used to reduce SiO_2_ to Si aerogel are aluminum (Al), calcium (Ca), magnesium (Mg), and lithium (Li). Compared to other metals, magnesium (Mg) works at low temperatures and maintains porosity, which is the structural form of aerogel and enables the production of silicon (Si).

The most efficient methods used for the reduction of SiO_2_ are the chemical etching method [25], chemical reduction process [26], chemical vapor deposition (CVD) [27,28], and template method [29]. These techniques each offer specific types of advantages and drawbacks. Templating allows for specific control of pore size and distribution, resulting in homogeneous structures that improve lithium-ion diffusion, although it may be challenging and costly. Although etching methods such as electrochemical or chemical etching are more manageable and more scalable, they frequently result in improperly controlled pore size and structural defects.

The MTR process has become a promising method for the conversion of porous templates into Si to make porous Si because of its low reaction temperature range (600~900 °C) [30,31,32]. Compared to other reduction processes, the MTR approach is very beneficial in converting porous silica aerogels (SiO_2_) to porous silicon (Si) while maintaining their porous structure, surface area, and low density, which is essential to enhancing lithium-ion battery efficiency [33]. This reduction involves the reaction of magnesium with silica, which results in an interlace composite product of magnesia (MgO) and silicon (Si) reactions.

The reduction reaction for SiO_2_ through magnesiothermic reduction is as follows:

SiO_2_(s) + 2Mg(g) → Si(s) + 2MgO(s) (1)

Mg(g) + Si(S) →Mg_2_Si(S)) + O_2_ (2)

SiO_2_(S) + 2MgO(S) →Mg_2_SiO_4_(S) (3)

Reducing SiO_2_ to Si by MTR, the achievable material not only takes benefit of silicon’s high theoretical capacity (3579~4200 mAh/g^−1^) but additionally reduces the mechanical degradation associated with silicon anodes [34].

In our study, we demonstrated a feasible approach to fabricate the porous silicon (Si) from mesoporous silica (SiO_2_) aerogel by the magnesiothermic reduction (MTR) method for the application of all-solid-state lithium-ion batteries (ASSLBs). MTR using hydrophobic and hydrophilic silica aerogel powder (SiO_2_) and magnesium powder (Mg) was observed at 600–900 °C. SiO_2_ could be converted into Si without significantly affecting its morphological structure. Increasing the temperature allowed Mg to vaporize. This vaporized magnesium then reacted with SiO_2_ to become Si while generating MgO, Mg_2_Si, and Mg_2_SiO_4_ as byproducts. The sample obtained after the reduction process was treated with hydrochloric acid (HCl) to remove byproducts. As prepared, porous silicon (Si) was characterized using various techniques. X-ray diffraction (XRD) was performed to identify the crystalline structure and phase composition of the aerogels, which revealed the successful production of Si. The material’s elemental composition was studied using X-ray fluorescence (XRF). Chemical bonding was observed by using Fourier transform infrared (FT-IR) spectroscopy. X-ray photoelectron spectroscopy (XPS) revealed significant details on the chemical states and surface composition, confirming the reduction of silica to silicon. Through scanning electron microscopy (SEM), we confirmed that the morphology and surface pore structure were maintained after the reduction of SiO_2_ to Si. Surface area analysis was also examined by using Brunauer–Emmett–Teller (BET) analysis. Furthermore, the coin cell was fabricated using Si as an anode, and the electrochemical performance was analyzed. The charge/discharge cycling tests revealed considerable improvements in capacity and stability, illustrating that porous Si has the potential to perform better than traditional anode materials. Our findings highlight the potential application of silicon (Si) aerogels produced from silica (SiO_2_) aerogels by MTR to improve ASSLB’s performance.

## 2. Results and Discussion

### 2.1. Formation Mechanism

In this study, two silica aerogel powders, hydrophobic and hydrophilic, were synthesized to clarify the effect of surface structure on MTR. The SiO_2_ aerogel undergoes a reduction process with magnesium (Mg), which converts SiO_2_ to Si via the reaction.

SiO_2_(s) + 2Mg(g) → Si(s) + 2MgO(s) (4)

**Figure 1:**
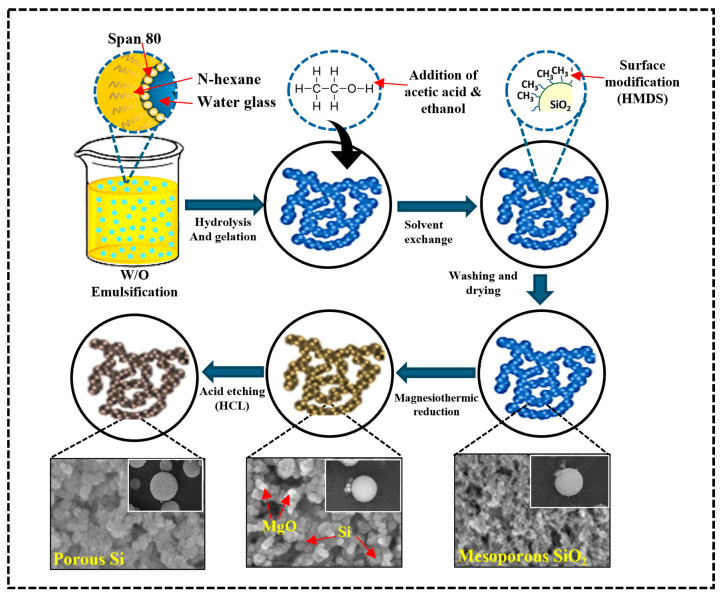
Formation mechanism of porous Si from mesoporous SiO_2_ aerogel.

During this process, the mesoporous structure of SiO_2_ is retained. The resulting Si-MgO composite was treated with hydrochloric acid (HCl) to eliminate the MgO byproduct, resulting in a porous Si with the mesoporous structure of the SiO_2_ aerogel with the chemical reaction. This approach has benefits in preserving high surface area and porosity, which are critical for applications that require significant interface interactions, such as lithium-ion batteries. Using SiO_2_ aerogel as a precursor for porous Si is particularly beneficial for lithium-ion batteries because of its high surface area and porous nature, which enhances the electrode’s capacity to absorb large volumes of lithium ions during charge and discharge cycles. Additionally, the mesoporous structure of Si provides effective paths for ion transport, resulting in increased electrochemical performance and stability in battery applications (Figure 1).

### 2.2. XRD Analysis

**Figure 2:**
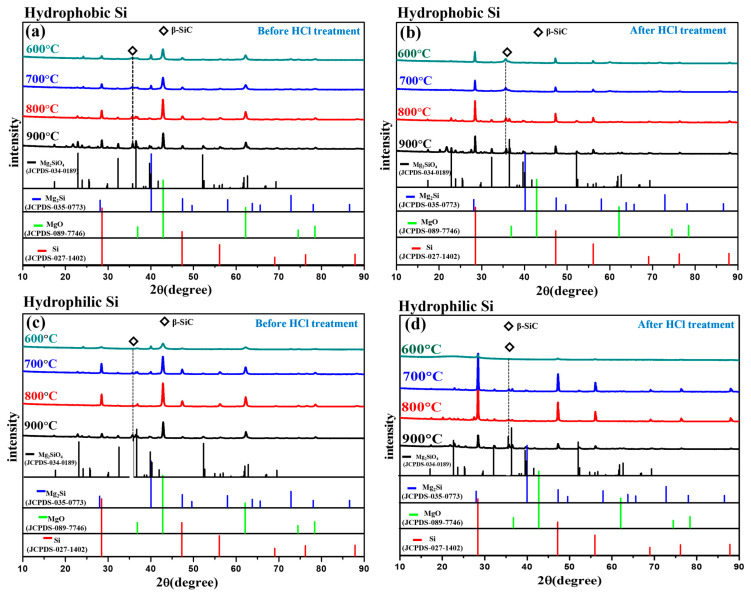
XRD pattern of porous silicon (Si) at reduction temperature 600~900 °C: (**a**,**b**) hydrophobic Si before and after HCL treatment; (**c**,**d**) hydrophilic Si before and after HCL treatment.

Figure 2 shows the X-ray diffraction (XRD) pattern of porous silicon (Si) prepared by MTR from SiO_2_ aerogel at different temperatures (600 °C, 700 °C, 800 °C, and 900 °C) to understand the phase evolution during the reduction process. A comparison of the XRD peaks before and after the HCl etching was carried out to ensure that the impurities were effectively removed following the treatment.

In Figure 2b, Si reduced from hydrophobic SiO_2_ revealed crystalline Si peaks at 2θ values of 28.4°, 32.34° 47.3°, 56.1°, 69.5°, and 76.5°, which correspond to standard diffraction peaks for silicon as per JCPDS Card No.027-1402. conversely, in Figure 2d Si prepared from hydrophilic SiO_2_ also showed crystalline Si peaks at 2θ values of 28.4°, 47.3°, 56.1°, 79.5°, and 76.5°. On the other side from Figure 2a,c, Before the HCl treatment, residual MgO (36.5°, 42.9°, 62.3°, 78.5°), Mg_2_Si (24.2°, 40.1°), and unreacted Mg_2_Si_4_ (17.44°, 20.12°, 21.74°, 22.09°, 25.38°, 27.54°, 52.22°) secondary phase peaks were observed in both hydrophobic and hydrophilic Si samples, which are consistent with JCPDS card numbers 089-7746, 035-0773, and 034-0189, respectively. These impurity peaks almost disappear after HCl treatment, indicating that MgO, Mg_2_Si, and Mg_2_Si_4_ were successfully removed by acid leaching. These results suggest that the HCl etching treatment effectively removes unwanted byproduct phases such as MgO and Mg_2_Si.

Mg(g) + Si(S) →Mg_2_Si(S)) + O_2_ (5)

**Figure 3:**
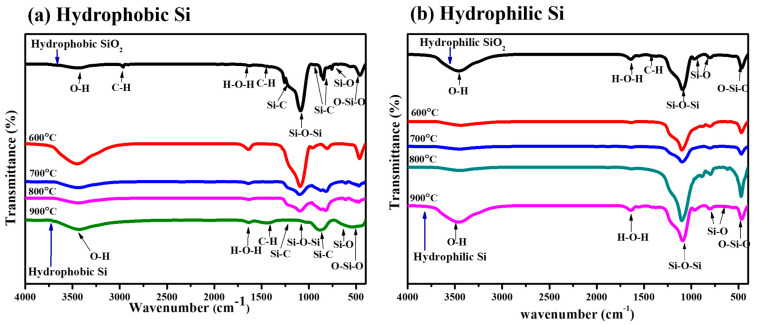
FT-IR spectra of (**a**) hydrophobic SiO_2_ and Si and (**b**) hydrophilic SiO_2_ and Si.

The interesting feature observed in the powder samples prepared from the hydrophobic and hydrophilic SiO_2_ is the formation of β-silicon carbide (SiC). The characteristic peaks at 35.6° correspond to β-SiC. It appears that the formation of β-SiC is due to the surface methyl groups (–(CH_3_)_3_) that are chemically bonded to SiO_2_ (–Si–O–). During the MTR, magnesium reduces the silica aerogel network structure to silicon according to Equation (1). At the same time, condensation reactions between –Si(CH_3_)_3_ and adjacent –Si(CH_3_)_3_ groups occur, leading to the formation of CH_4_ and H_2_ from the consumed CH_3_ groups [35]. The resulting CH_4_ gas subsequently reacts with silicon to form SiC (Si + CH_4_ → SiC + 2H_2_). Generally, the crystalline β-SiC formation begins above 1400 °C via a gas phase reaction between SiO and CO, as is typically observed in the carbothermal reduction of silica. The presence of magnesium vapor during MTR plays a crucial role in facilitating the formation of SiC. Further confirmation of this transformation can be seen in the FT-IR spectra shown in Figure 3 [36].

For hydrophobic and hydrophilic SiO_2_, a comparison analysis of the Si XRD peaks across the reduction temperature range of 700 °C to 800 °C shows a constant rise in peak intensity with rising temperature. At a reduction temperature of 800 °C, the highest peak intensity of Si was observed, indicating exceptional crystallinity for both forms of SiO_2_ at this temperature. However, at a higher temperature of 900 °C, moderate MgO and Mg_2_SiO_4_ peaks indicate that excessive thermal energy affected the secondary reaction involving residual Mg, SiO_2_, and O_2_. As a result, higher temperatures increased the Si crystallinity and the possibility of secondary phase development.

### 2.3. XRF Analysis

As shown in Table 1 above, an X-ray fluorescence analysis (XRF) study of the MTR of Si from SiO_2_ (hydrophobic/hydrophilic) at different temperatures reveals elemental composition. For hydrophobic Si, the percentage of silicon (Si) increased to 60.95% with the increasing temperature. The oxygen (O) content decreased to 24.76% simultaneously, indicating that the reduction of SiO_2_ to silicon was more effective at 800 °C. However, as the temperature increased over 800 °C, the oxygen concentration of the 900 °C treated sample increased compared to the 800 °C sample, indicating that the reduction process was incomplete. In addition, the magnesium (Mg) content rose with the temperature, peaking at 8.74% at 900 °C, demonstrating magnesium’s significant role in reduction at high temperatures.

In comparison to hydrophobic Si, hydrophilic Si showed a higher temperature dependency during silicon reduction. The silicon concentration was 45.41% at 600 °C, but it increased rapidly as the temperature rose, reaching 75.87% at 800 °C. Alongside the increase in silicon concentration, the oxygen percentage decreased to 15.29%. This indicates that SiO_2_ is reduced more effectively at higher temperatures under hydrophilic conditions. Increasing the temperature also affected the concentration of magnesium, which was observed at 900 °C (Mg at 14.42%). The rise in magnesium concentration indicates that it plays an active role in the reduction process [37].

XRF analysis reveals that, at 800 °C, the maximum silicon content was produced in both hydrophobic and hydrophilic conditions, with hydrophilic conditions showing a more effective reduction. In both situations, the ideal temperature for reduction was 800 °C. These findings highlight the importance of surface characteristics and temperature for MTR for improved silicon production.

### 2.4. FT-IR Analysis

The FT-IR spectra in Figure 3a,b show structural changes in (hydrophobic/hydrophilic) silica (SiO_2_) and silicon (Si) during MTR at temperatures ranging from 600 °C to 900 °C. The (hydrophobic/hydrophilic) SiO_2_ samples showed peaks between 1080 cm^−1^ and 800 cm^−1^, confirming Si-O-Si asymmetric stretching and bending vibrations [38]. As the temperature rose, these peaks eventually decreased, indicating the breakdown of the silica framework and its reduction to Si. The successful elimination of hydroxyl (–OH) groups can be observed by the absence of a wide peak at 3200–3600 cm^−1^. New peaks at 470 cm^−1^ and 615 cm^−1^ reveal Si-Si stretching and bending vibrations, demonstrating the formation of crystalline silicon. Significantly, as explained in the previous discussion at a higher temperature of 900 °C, the wide peak at 3200–3600 cm^−1^ that represents hydroxyl (–OH) groups grew because of secondary phase development of residual Mg, SiO_2_, and O_2_ caused due to excessive thermal energy. In the above discussion, XRD confirmed β-SiC production, but only the hydrophobic sample showed the Si-C peak in FTIR. This could be owing to low surface concentration or overlap with significant Si-O-Si absorption in the hydrophilic sample.

In conclusion, SiO_2_ is present at lower temperatures, indicated by Si-O-Si peaks, which suggests that reduction is incomplete. Increasing the temperature helps to achieve silicon, but at a certain point, the reappearance of Si-O-Si peaks indicates that silicon has partially reoxidized or developed of secondary phase. This highlights the importance of temperature control throughout the reduction.

### 2.5. XPS Characterization

**Figure 4:**
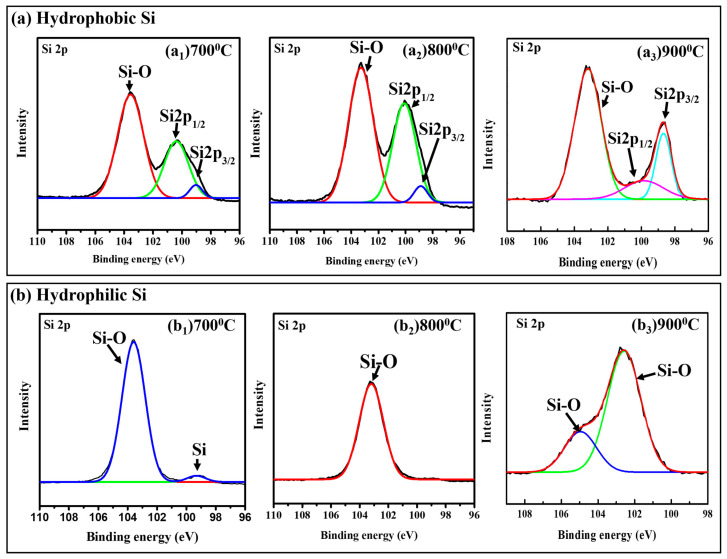
XPS spectrum of Si 2p from reduced Si: (**a**) hydrophobic Si ((**a_1_**)—700 °C, (**a_2_**)—800 °C, (**a_3_**)—900 °C) and (**b**) hydrophilic Si ((**b_1_**)—700 °C, (**b_2_**)—800 °C, (**b_3_**)—900 °C).

The X-ray photoelectron spectroscopy (XPS) spectra of Si 2p of hydrophobic and hydrophilic Si at temperatures (700 °C, 800 °C, and 900 °C) are shown in Figure 4a_1_–a_3_ and Figure 4b_1_–b_3_, respectively. For calibration, a C-C binding modification at 284.8 eV was used. The Si 2p peaks were studied to investigate the elemental composition and bonding phases of the Si surfaces, highlighting the temperature-dependent oxidation and chemical environment changes.

As shown in Figure 4a_1_–a_3_, all Si 2p peaks were deconvoluted in three different peaks: the peak situated at 99.3 eV (related to the Si-Si metallic bond), the second peak at 101.0 eV ascribed to the Si-C bond, and the third peak located at 103.0 eV related to the Si-O/Si-O-C bond formation [39]. With increasing reaction temperature, the peak intensity was increased, as shown in Figure 4a_1,_a_2_. Whereas, at a reaction temperature of 900 °C (Figure 4a_3_,) the peak intensity decreased, which was mainly due to an elevated reaction temperature (900 °C). Excessive thermal energy affects secondary reaction involving residual MgO and Mg_2_SiO_4_ formation. Because of the passivated surface, which prevents oxidation until higher temperatures rise above its stability, the Si 2p spectra of hydrophobic silicon show temperature-dependent oxidation.

Conversely, due to its strong surface reactivity, hydrophilic silicon’s Si 2p spectra in Figure 4b_1_–b_3_ show rapid oxidation. The peaks for SiO and SiO_2_ at ~99.3 eV (Si-Si) and ~103.0 eV (Si-O) at 700 °C (Figure 4b_1_) show significant oxidation with minimal elemental Si remaining. The SiOx peak increased, indicating moderate oxidation, and the SiO_2_ peak grew more intense, indicating a denser oxide layer at 800 °C (Figure 4b_2_). At 900 °C (Figure 4b_3_), the observed peak at 105 eV is ascribed to a higher oxidation state silicon atom (Si^4+^), which is mainly formed due to the oxidation of the SiO material at high temperatures [40].

High-resolution XPS C 1s spectra of hydrophobic and hydrophilic Si samples (800 °C) were studied to detect the presence of Si-C bonds as Supplementary Materials (Figure S1). In Figure S1a, the hydrophobic Si sample has peaks at 282.8 eV, 284.2 eV (C-C/C-H), and 285.6 eV (O-C=O), with one peak slightly touching the Si-C binding energy range (283.0–283.5 eV). The hydrophilic sample in Figure S1b has peaks at 284.8 eV (C-C/C-H), 285.8 eV (O-C=O), and 288.4 eV (C-O) but no Si-C-related peak. The relatively small peak in the hydrophobic sample can be attributed to surface modification (CH_3_). However, the absence of a peak in the hydrophilic sample has no surface modification, highlighting the detection limit. Additionally, the above FTIR spectra (Figure 3a,b) indicate no absorption near 1250 cm^−1^ in the hydrophilic sample and only a weak peak in the hydrophobic sample, supporting the low presence of Si–C bonds.

The XPS results show that oxidation depends on temperature and surface characteristics—hydrophobic silicon resists oxidation until higher temperatures, while hydrophilic silicon oxidizes more readily. Although XRD confirms β-SiC formation in both samples, a weak peak of the Si–C signal appeared in XPS, likely due to its limited surface sensitivity. A similar trend is seen in FTIR, where Si–C vibration was only detected in the hydrophobic sample. These results confirm that Si–C bonds are either absent or present in negligible amounts below the detection limits in both XPS and FTIR analyses. This suggests that in the hydrophilic sample, β-SiC mainly exists in the bulk, beyond the detection range of FTIR and XPS, but is observable by XRD.

### 2.6. SEM Characterization

The scanning electron microscopy (SEM) study of reduced hydrophobic and hydrophilic SiO_2_ to Si at various temperatures (700–900 °C) demonstrates the structural and morphological changes induced by HCl treatment. The overall morphology of hydrophobic and hydrophilic SiO_2_ has been investigated in previous studies [41,42], and reduced Si before and after MTR and subsequent acid (HCl) treatment was observed. The resulting pure silicon (Si) exhibits spherical morphology and a porous surface. The impact of different temperatures (700–900 °C) on the surface characteristics and particle size of SiO_2_ and Si particles was thoroughly examined.

**Figure 5:**
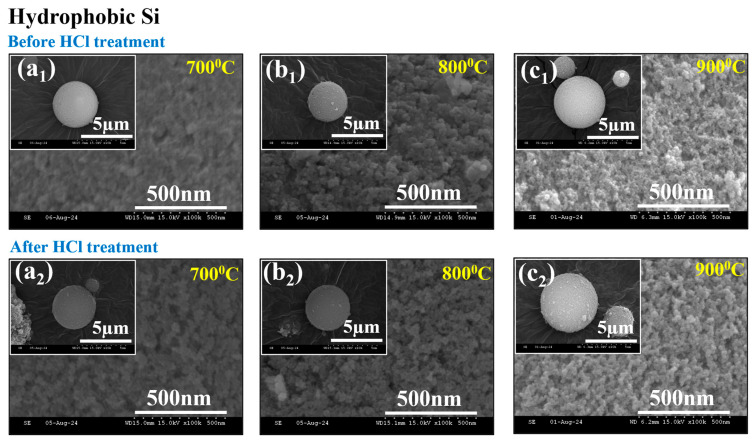
SEM analysis of reduced Si (hydrophobic) before and after HCl treatment at different temperature variations—(**a_1_**,**a_2_**) 700 °C, (**b_1_**,**b_2_**) 800 °C, (**c_1_**,**c_2_**) 900 °C.

Figure 5 shows The SEM micrographs of reduced (hydrophobic) Si before/after HCL treatment with 700 °C, 800 °C, and 900 °C reduction temperatures. Figure 5a_1_,a_2_ show the morphology and surface of reduced Si at 700 °C before and after HCl treatment. After reduction, the presence of MgO on the Si surface resulted in a denser surface, although the spherical shape remained unchanged. On the other hand, after HCl etching, well-maintained spherical microspheres and reduced pore distribution took place due to the aggregation of surface particles. At a higher temperature of 800 °C and 900 °C in Figure 5b_1_,b_2_,c_1_,c_2_, similar results are observed, where the surface exhibits a porous morphology with spherical microspheres, and after HCl treatment, there was a slight reduction in pore size due to particle aggregation, but the shape of the particles remained unchanged even at high temperatures and after acid etching.

Additionally, the Supplementary Materials (Figure S2) shows SEM images and corresponding particle size distributions of hydrophobic SiO_2_ and Si particles at a reduction temperature of 800 °C both before and after HCl treatment. The hydrophobic SiO_2_ particles exhibit a uniform spherical morphology with a relatively small size distribution (3.543 µm). Following thermal reduction, the hydrophobic Si particles show a slight increase in size (4.527 µm) and surface roughness, likely due to partial particle coalescence at elevated temperatures. After HCl treatment, the spherical morphology was retained, while the particle size distribution slightly decreased (3.4943 µm), indicating the effective removal of surface residues and improve uniformity. Overall, temperature variation did not have much impact on the particle size, with only minor differences observed.

**Figure 6:**
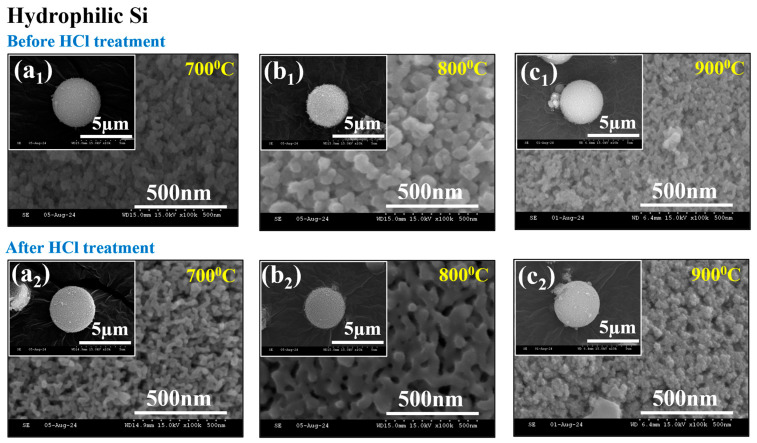
SEM analysis of reduced Si (hydrophilic) before and after HCl treatment at different temperature variations—(**a_1_**,**a_2_**) 700 °C, (**b_1_**,**b_2_**) 800 °C, (**c_1_**,**c_2_**) 900 °C.

To confirm the spherical morphology and porous surface of Si (hydrophilic) prepared through MTR, SEM analysis was conducted, as shown in Figure 6. Initially, Figure 6a_1_,a_2_ demonstrate that after reduction at 700 °C, the reduced Si particle appeared to have a denser and more compact surface. However, after HCl treatment, the structure became more porous due to the removal of MgO/Mg_2_SiO_4_ byproducts formed during MTR with maintained morphology. However, Figure 6b_1_,b_2_ show enhanced porosity observed because of enhanced etching at 800 °C. At 900 °C, complete sintering took place, and slightly decreasing porosity was observed, as shown in Figure 6c_1_,c_2_. HCl treatment improved the surface area and pore dispersion, while magnesium (Mg) acted as both a reducing agent and a template simultaneously.

The SEM study indicates that the spherical shape and porous surface of Si produced by MTR were well preserved after HCl treatment. The increased temperature slightly caused further aggregation and pore reduction due to the sintering effect and enhanced diffusion of MgO into the Si matrix, resulting in a denser structure. These findings highlight the porous silicon structure’s ability under varying thermal and chemical treatments.

### 2.7. BET Characterization

**Figure 7:**
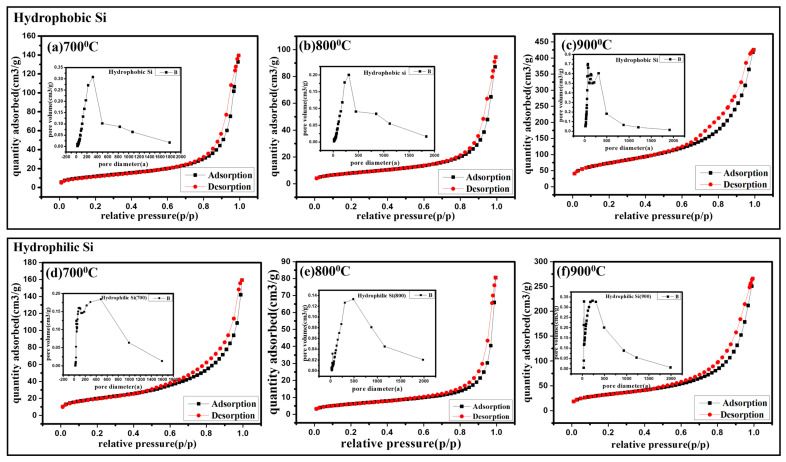
BET surface area analysis of hydrophobic Si—(**a**) 700 °C, (**b**) 800 °C, (**c**) 900 °C—and hydrophilic Si—(**d**) 700 °C, (**e**) 800 °C, (**f**) 900 °C. Insets show pore size distribution based on BJH (adsorption).

To examine the temperature-dependent effects on the pore size distribution, pore volume, and surface area of hydrophobic and hydrophilic Si, N_2_ adsorption–desorption experiments were conducted. The obtained results are displayed in Figure 7a–f. The N_2_ adsorption–desorption isotherms of all six samples exhibited a characteristic type IV isotherm and an H3-type hysteresis loop at high pressure, confirming the presence of mesopores, as shown in Figure 7a–f [43]. Table 2 presents the calculated Brunauer–Emmett–Teller (BET) specific surface area (SSA), average pore volume, and pore size distribution values for hydrophobic and hydrophilic Si.

Temperature variation significantly impacted the structure of hydrophobic Si. As shown in Figure 7a,b, adsorption increased slightly between 700 °C and 800 °C, indicating stable mesopores. However, Figure 7c reveals a sudden rise at 900 °C, suggesting the formation of smaller pores. This aligns with the BET results, which show a decrease in pore size and an increase in surface area, likely because of elevated temperatures and pore restructuring. Figure 7d shows that the adsorption curve at 700 °C for hydrophilic Si indicates a well-defined porous structure. However, at 800 °C, as shown in Figure 7e, a noticeable decrease in surface area occurred due to the collapse of the pore network. At 900 °C, like the trend observed for hydrophobic Si (Figure 7c), increasing temperature significantly enhanced the surface area. The BJH pore size distribution further supports this, showing that at 900 °C, the pore size of hydrophilic Si decreases while maintaining a stable mesoporous framework, whereas hydrophobic Si at the same temperature develops finer pores [44].

Temperature-dependent changes in surface area, pore volume, and pore size were discussed in Table 2. For hydrophobic Si, at lower temperatures (700 °C to 800 °C), structural densification led to a reduction in surface area (43.85 m^2^/g to 29.29 m^2^/g) and pore volume (0.211 cm^3^/g to 0.144 cm^3^/g), likely due to particle agglomeration and partial collapse of the porous framework. However, at 900 °C, a substantial increase in surface area (267.1 m^2^/g) and pore volume (0.634 cm^3^/g) occurred, indicating the formation of new pores. This transformation was accompanied by a significant reduction in pore size from 19.55 nm at 800 °C to 10.56 nm at 900 °C, highlighting a shift from mesoporous to micro-mesoporous structures. For hydrophilic Si, the trend followed a similar pattern of temperature-dependent changes but with notable differences in the porous network’s behavior. At lower temperatures (700 °C and 800 °C), the material underwent surface area loss due to pore collapse and a reduction in the overall porosity. However, at higher temperatures (900 °C), the material’s surface area stabilized, and fine mesopores were formed, contributing to a more interconnected porous network.

The BET study suggests significant structural changes in both hydrophobic and hydrophilic Si as the temperature increases. For hydrophobic Si, at lower temperatures, the densification of particles affects reducing the surface area and pore volume. At higher temperatures, mesopore development causes a dramatic rise. On the other hand, hydrophilic Si shows a more interconnected porous network when it comes to higher temperatures after experiencing a surface area loss at lower temperatures. These findings highlight how porosity development can be affected by temperature variation, which is important for tailoring material properties for specific applications.

### 2.8. Electrochemical Characterization of Porous Si Anode

#### 2.8.1. Schematic Representation of Half-Cell Assembly for Silicon-Based Anode

**Figure 8:**
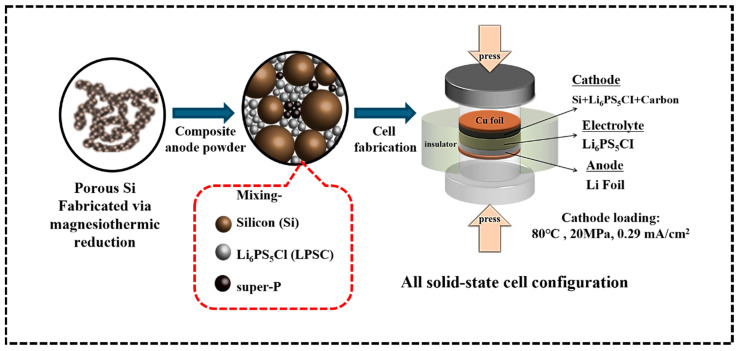
Schematic diagram of half-cell assembly for magnesiothermic reduced silicon-based anode and solid electrolyte.

As seen in Figure 8, the fabrication mechanism of an all-solid-state battery involves key steps to ensure effective ion and electron flow. An argyrodite-type sulfide [Li_6_PS_5_Cl (LPSC)] with strong ionic conductivity up to 1.5 × 10^−3^ is pressed into a pellet at 100 MPa, resulting in a dense electrolyte layer. The (Si:LPSC:Super-P) composite electrode, with a ratio of 70:30:10, balances electronic conductivity (Super-P) and ionic conductivity (LPSC), while silicon is the active material. The Li foil electrode and Cu current collectors provide adequate electron flow. Pressing an entire cell at 20 MPa improves interfacial contact and reduces resistance. During charge/discharge cycles (0.02–2 V vs. Li, 80 °C, 0.29 mA/cm^2^), the solid electrolyte allows fast Li-ion transport. At the same time, the composite electrode structure supports stable lithiation and delithiation of silicon, which is crucial for battery performance.

#### 2.8.2. Electrochemical Performance

**Figure 9:**
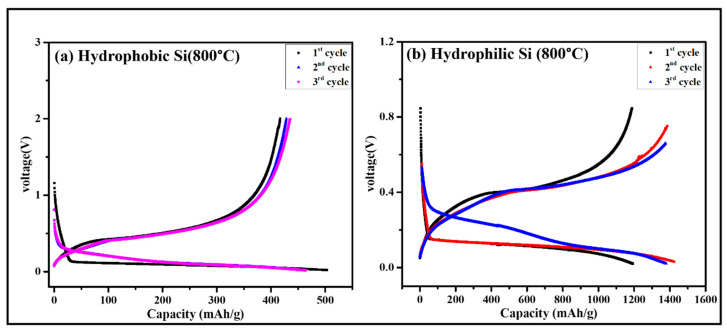
Charge/discharge curve of (**a**) hydrophobic Si and (**b**) hydrophilic Si anode for ASSCs with the composition of Si:LPSC:C.

The charge–discharge curves of hydrophobic and hydrophilic silicon (Si)-based composite electrodes for ASSCs are shown in in Figure 9, and the higher silicon content effect on half-cell impedance resistance and conductivity is discussed in the Supplementary Materials (Figure S3). For electrochemical testing, samples at the 800 °C reduction temperature having higher silicon percentages (60.75% for hydrophobic and 75.87% for hydrophilic) were used. The electrodes were examined under equal conditions: a Si:LPSC:Super-P composite ratio of 70:30:10 with Li foil as the counter electrode, Cu as the current collector, a voltage range of 0.02–2 V (vs. Li) at 80 °C, and a current density of 0.29 mA/cm^2^.

As shown in Figure 9a, the initial discharge capacity of the hydrophobic Si electrode was 503.2 mAh·g^−1^; however, higher polarization can be detected in the voltage profiles, which is caused by the solid electrolyte’s poor interfacial contact with the hydrophobic Si. Resistance increases because of this poor interface, restricting Li-ion transport and decreasing capacity retention and Coulombic efficiency across cycles. In the second cycle, the capacity decreased to 454.7 mAh·g^−1^, and in the third cycle, it slightly improved to 465.9 mAh·g^−1^. Poor interfacial contact and the less hydrophobic Si content (60.75%) limit the electrochemical electrode’s overall performance by decreasing the total amount of active sites available for Li-ion storage.

On the other hand, compared to hydrophobic Si electrodes, the electrochemical performance of the hydrophilic Si electrode was improved. As shown in Figure 9b, the first cycle discharge capacity was 1190.5 mAh·g^−1^, which is nearly double the capacity of the hydrophobic Si electrode. The voltage profiles indicate decreased polarization, indicating improved wettability and interfacial compatibility between the hydrophilic Si and the solid electrolyte. Additionally, a higher amount of active material is provided by the hydrophilic electrode’s higher silicon content (75.87%), which helps to improve capacity. Possibly because of more Li-ion storage sites being activated, the capacity rose to 1423.0 mAh·g^−1^ in the second cycle and stabilized at 1379.4 mAh·g^−1^ in the third cycle, indicating improved cycling stability.

Electrochemical impedance spectroscopy (EIS) was conducted on all-solid-state cells incorporating hydrophilic and hydrophobic silicon, as shown in the Supplementary Materials (Figure S3). The hydrophilic silicon half-cell, containing a higher silicon content, exhibited a lower impedance resistance (27.34 Ω) than the hydrophobic (33.16 Ω). This suggests that the increased silicon content in the hydrophilic sample contributes to improved ionic transport and reduced interfacial resistance within the cell. These findings highlight the role of surface properties and silicon content in optimizing cell performance. The hydrophilic silicon half-cell, with higher silicon content, demonstrated lower interfacial resistance, indicating enhanced ionic transport in all-solid-state configurations.

Also, for a better understanding of the charge/discharge cycle performance, samples containing 5 wt% hydrophilic Si and 95 wt% graphite were examined to evaluate composite performance. Following the initial cycles at 0.1 C, the cell was cycled at 0.1 C for 100 cycles (0–1.5 V). The initial discharge capacity exceeded 426.96 mAh/g and maintained greater than 382.34 mAh/g after 100 cycles, indicating consistent performance. The capacity retention after 100 cycles was 89.5%. The voltage capacity profile resembles conventional graphite behavior, demonstrating effective lithium intercalation. Despite the low active material content, its impact was significant, indicating that graphite anodes can be improved even at modest loadings. Full electrochemical data and cycling stability are provided in the Supplementary Materials (Figure S4).

The specific capacities of hydrophobic and hydrophilic Si electrodes during three cycles were compared in Table 3. In the first cycle, the discharge capacity of the hydrophobic electrode was 503.2 mAh·g^−1^, which decreased to 454.7 mAh·g^−1^ in the second cycle and improved slightly to 465.9 mAh·g^−1^ in the third cycle. These results illustrate the issues caused by the low silicon content (60.75%) and poor wettability, which lead to increased interfacial resistance and less Li-ion storage. But the hydrophilic electrode has a much greater first cycle discharge capacity of 1190.5 mAh·g^−1^. The capacity increases to 1423.0 mAh·g^−1^ in the second cycle while stabilizing at 1379.4 mAh·g^−1^ in the third cycle. The increased efficiency is due to a combination of its higher silicon content (75.87%) and improved wettability, which will enhance interfacial contact with the solid electrolyte, reduce resistance, and allow for effective Li-ion conduction. The more active sites for Li-ion storage are available because of the high Si percentage.

These results demonstrate the importance of material characteristics, mainly silicon concentration, surface wettability, and interfacial compatibility, which affect the performance of Si-based composite electrodes for ASSLBs [45]. The hydrophilic Si electrode, with an optimal combination of these properties, has higher capacity and cycle stability, which makes it the preferred option for ASSLB application.

## 3. Conclusions

In summary, a porous silicon (Si) anode for all-solid-state lithium-ion batteries (ASSLBs) was successfully developed using magnesiothermic reduction (MTR) of mesoporous silica (SiO_2_) aerogel. This method was effective for preserving the Si structure and improving its properties, such as surface area, pore size, and electrochemical performance. Characterization methods such as FT-IR, XRD, XRF, XPS, SEM, and BET confirmed the effects of temperature and magnesium impact on silicon’s reliability and performance. Better stability and a longer cycle life have been obtained by optimizing the reduction process to produce porous silicon, which is capable of volume variations during lithiation and delithiation.

In this work, hydrophobic and hydrophilic silica were used to produce both types of silicon. Both kinds of silicon were used to study noticeable differences in their performance and properties. The findings provide more insight into the development of materials optimization for specific applications by highlighting the effects of hydrophobic and hydrophilic properties on pore size and surface area, and additionally, the results showed the importance of surface area and pore size in achieving optimal electrochemical performance. The MTR process is superior to other methods in maintaining material properties and improving the wettability and compatibility of silicon. Coin cell tests for three cycles at a 1 C rate, 0.02–2 V, and 100 cycles at a 1 C rate, 0–1.5 V, confirmed the Si material anode’s practical potential using Si:LPSC:Super-P composite, with stable charge and discharge cycles. This study demonstrates the correlation between the material properties and battery performance, which offers essential details for designing durable and high-capacity anodes for ASSLBs. MTR is a scalable and efficient technique that produces high-quality silicon at lower temperatures and in less time. Future research could focus on increasing silicon content, improving long-term performance, and optimizing the interface between the anode and electrolyte to make these batteries even more helpful for wide applications.

## 4. Materials and Methods

### 4.1. Materials

The reagents to synthesize silica (SiO_2_) aerogel and for the reduction silicon(Si) were used as follows: water glass (silica content: 28–30 wt.%, SiO_2_/Na_2_O = 3.4:1, Young Il Chemical Co., Ltd., Incheon, Republic of Korea), N-hexane (95%, Samchun Pure Chemical, Seoul, Republic of Korea), acetic acid (99.5%, Samchun Pure Chemical, Pyeongtaek, Republic of Korea), Ethyl alcohol (95.0%, Samchun Pure Chemical, Republic of Korea), and hexamethyldisilazane (HMDS, 98%, Samchun Pure Chemical, Korea). Sorbitan monooleate (Span80, Junsei Chemical Co., Ltd., Tokyo, Japan), 2-Propanol (95%, Samchun Pure Chemical, Seoul, Republic of Korea), magnesium powder (Mg, 98%, 20–230 mesh, Sigma Aldrich, Darmstadt, Germany), hydrochloric acid (HCl, 35–37%, Junsei Chemical, Japan). The solid electrolyte (LPSC) and Li metal foil were purchased from Posco JK solid solution (Pohang-si, Republic of Korea).

### 4.2. Method

#### 4.2.1. Fabrication of Mesoporous Silica (SiO_2_) Aerogel Powder

**Figure 10:**
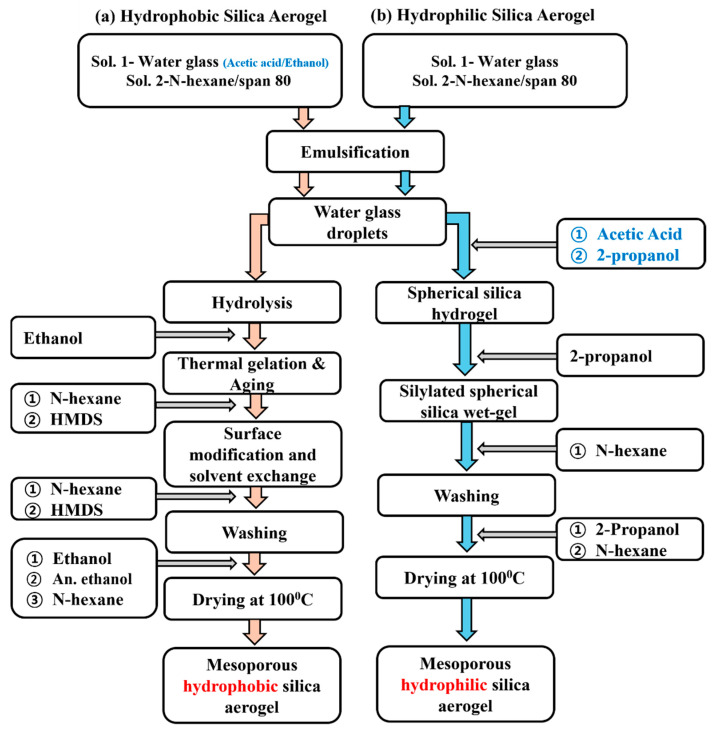
Experimental flow chart for the synthesis of spherical (**a**) hydrophobic and (**b**) hydrophilic silica (SiO_2_) aerogel.

Spherical hydrophobic silica aerogel (SiO_2_) powder was fabricated using thermal gelation [41]. As shown above in Figure 10a, a brief procedure was conducted as follows: a water glass sodium silicate solution was used as the initial precursor of silica. Deionized water was mixed with sodium silicate to make 8.68 wt.% of water glass solution. Next, ethyl alcohol and acetic acid were mixed in a 75 mL water glass. Subsequently, 85 mL of N-hexane was mixed with the surfactant sorbitan monooleate (span 80). The ratio between water glass to n-hexane was maintained at 1:1. N-hexane/water glass solution was homogenized for 10 min at 6000 rpm (UltraTurrax IKA T25:S25D-10G-KS; IKA Werke, Königswinter, Germany). For the condensation (thermal gelation), the resulting water glass/n-hexane solution was heated to 100 °C. After 90 min, wet silica gel was immersed in 150 mL of ethyl alcohol. A solvent exchange agent (ethyl alcohol) could affect the hydrogel-to-alcogel transition. An amount of 150 mL of 20% hexamethyldisilazane (HMDS) was used for chemical modification of spherical silica aerogel surface for three hr. of continuous stirring at 100 °C. The silylated silica wet gel spheres were washed with an ethyl alcohol/n-hexane solution to eliminate any residual surface modification agents and reaction products. The silica wet gel spheres were dried at 100 °C under ambient pressure for 1 h. Further investigations and characterization were conducted using the obtained hydrophobic silica (SiO_2_) aerogel.

Following the synthesis of spherical hydrophilic silica aerogel (SiO_2_), the emulsion polymerization method was used to synthesize hydrophilic silica (SiO_2_) aerogel [42]. Figure 10b demonstrates the experimental flow chart for the synthesis of the hydrophilic silica (SiO_2_) aerogel. Water glass sodium silicate was used as a precursor of silica solution and diluted into deionized water to make 11.7 wt.% water glass solution. Initially, 75 mL of water glass solution was prepared and then mixed with 80 mL n-hexane solution containing sorbitan monooleate (span 80) surfactant. The water glass-to-n-hexane ratio remained constant at 1:1. Next, the n-hexane/water glass solution was mixed by using a homogenizer (UltraTurrax IKA T25:S25D-10G-KS; IKA Werke, Königswinter, Germany) at 6000 rpm for 10 min. After homogenization, acetic acid and 150 mL of 2-propanol were added to the resulting emulsion solution for hydrolysis of water glass at room temperature. After one hour, the very first 150 mL of 2-propanol was removed from the wet silica gel, and an equal volume of new 2-propanol was added, and the solution was treated at 100 °C for 1 h with continuous stirring. The addition of 2-propanol led to gelation and aging of silica gel. After 1 h of continuous stirring and heating, reacted 2-propanol was replaced with n-hexane to reduce the surface tension and capillary forces, preventing pore collapse during the drying process. The silica wet gel spheres were washed with 2-propanol/n-hexane solution to eliminate residue agents and reaction products. After washing, the collected silica wet gel was dried under ambient pressure conditions at 100 °C. The obtained hydrophilic silica (SiO_2_) aerogel was used for further experimentation and characterization.

#### 4.2.2. Magnesiothermic Reduction of Mesoporous SiO_2_ to Porous Si

**Figure 11:**
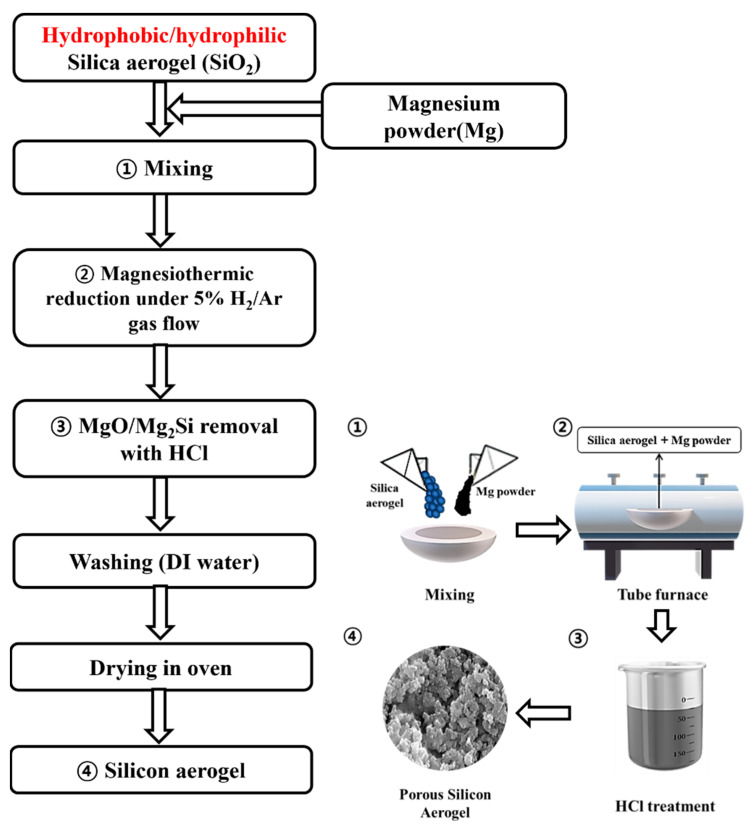
Experimental flow chart for the magnesiothermic reduction (MTR) of mesoporous SiO_2_ to porous Si.

The synthesized mesoporous (hydrophobic/hydrophilic) silica (SiO_2_) aerogels have been converted into porous silicon (Si) via MTR [46]. Figure 11 shows a detailed illustration of the MTR process, which noticeably outlines each step. In brief, prepared SiO_2_ was physically mixed with magnesium (Mg) powder in a 1:2 molar ratio for 10 min to ensure uniform distribution of the materials. After that, the mixture was placed in a tube furnace using an alumina boat. Afterward, a constant temperature increase rate of 5 °C min^−1^ was applied to a temperature of 600~900 °C to enable the reduction process of SiO_2_ in the presence of a 5% H_2_/Ar gas environment. The reaction was allowed to proceed for 4 h, and after the reaction, impurities such as MgO and Mg_2_Si generated through side reactions between Si and Mg were removed by reacting with 2 M hydrochloric acid (HCl) with continuous stirring for 6 h. The solutions were filtered 2 times using DI water and dried in an oven at 100 °C.

### 4.3. Characterization

The crystal structure of the samples prepared under different temperatures was analyzed using X-ray diffraction analysis (XRD-Smart Lab SE, Standard Analysis Research Institute, Inha University, Incheon, Republic of Korea). X-ray fluorescence analysis (XRF- ZSX Primus IV, Hanyang University Seoul Joint Equipment Center) was used to determine the elemental composition of materials, providing precise quantitative data. During the MTR of SiO_2_ to Si, functional groups were identified, and chemical bonding changes were confirmed using Fourier transform infrared spectroscopy (FT-IR-VERTEX 80V, Standard Analysis Research Institute, Inha University, Incheon, Republic of Korea) was conducted. The elements and components on the surface of the prepared samples were examined using X-ray photoelectron spectroscopy (XPS, K-Alpha, Standard Analysis Research Institute, Inha University, Incheon, Republic of Korea). Field emission scanning electron microscopy (FE-SEM, Hitachi S-4300, Sustainable Energy Components and Materials Core Research Support Center, Inha University, Incheon, Republic of Korea) was used to compare the surface morphology and microstructure after/before HCl treatment of reduced Si. The surface area and pore distribution were determined by Specific Surface Analyzer II (BET-3Flex, Standard Analysis Research Institute, Inha University, Incheon, Republic of Korea) analysis from the amount of N_2_ gas adsorbed.

### 4.4. Electrochemical Measurement

#### All-Solid-State Cell (ASSC) Configuration

To examine the electrochemical performance of the produced Si, an all-solid-state cell (ASSC) was assembled in an argon-filled glove box using it as an electrode material. A 10 mm diameter mold was filled with a solid electrolyte based on argyrodite-type sulfide Li_6_PS_5_Cl (LPSC) powder and uniaxially pressed at 100 MPa to make a pellet. The powder composite anode composed of Si with Si:LPSC:super-P composite anode with a ratio of 70:30:10. Li foil was placed on the opposite solid electrolyte side to serve as a cathode, and Cu foil was placed as the current collector of both electrodes. Afterward, the entire cell was pressed at 50 MPa to make an all-solid-state battery of Si, LPSC/super-P/Lhi foil. To conduct a charge/discharge experiment on the assembled ASSCs, conductors connected to the battery test system (VMP-300) were connected to both terminals of the cell, and the charge/discharge conditions were 0.02~2V (vs. Li), 80 °C, and 0.29 mA/cm^2^. The temperature was maintained using a PTFE heating jacket and mini SQ heating controller during the test. At this time, all charging and discharging conditions were conducted at constant current.

To further validate and evaluate the practical applicability of the reduced Si in a composite anode, a sample containing 5 wt% reduced Si and 95 wt% graphite was tested with cycling over 100 cycles at 1 C rate, 0–1.5 V (vs. Li), 25 °C.

## Figures and Tables

**Table 1:** XRF analysis of Si obtained from hydrophobic and hydrophilic SiO_2_ at different temperatures.

Sample	Temperature (°C)	Si (wt.%)	O (wt.%)	C (wt.%)	Mg (wt.%)
Hydrophobic Si	600	55.64	30.51	10.50	2.91
	700	56.61	26.03	12.97	3.83
	800	60.95	24.76	7.98	6.16
	900	42.51	42.00	6.27	8.74
Hydrophilic Si	600	45.41	49.22	4.63	0.17
	700	64.74	23.26	5.15	5.74
	800	75.87	15.29	4.22	3.47
	900	42.33	39.18	2.40	14.42

**Table 2:** BET surface area and pore size analysis of Si obtained from hydrophobic and hydrophilic SiO_2_.

Sample	Temperature (°C)	Surface Area (m^2^/g)	Pore Volume (cm^3^/g)	Pore (Desorption) Size (Å)	Mean Pore Diameter (nm)
Hydrophobic Si	700	43.85	0.211	194.2	19.42
	800	29.29	0.144	195.5	19.55
	900	267.1	0.634	105.6	10.56
Hydrophilic Si	700	72.80	0.240	134.0	13.40
	800	22.48	0.123	213.1	21.31
	900	118.1	0.404	142.4	14.24

**Table 3:** Comparison of electrochemical performance (first three cycles) between hydrophobic and hydrophilic Si electrodes.

Sample	Hydrophobic Si	Hydrophilic Si
Reduction temp. (°C)	800	
Composite ratio	70:30:10	
1st cycle capacity (mA·h·g^−1^)	503.2	1190.5
2nd cycle capacity (mA·h·g^−1^)	454.7	1423.0
3rd cycle capacity (mA·h·g^−1^)	465.9	1379.4

## Data Availability

All data and materials are available upon request from the corresponding author. The data are not publicly available due to ongoing research using a part of the data.

## Supplementary Materials

The following supporting information can be downloaded at https://www.mdpi.com/article/10.3390/gels11040304/s1. Figure S1: XPS spectrum of C1 S from reduced Si (a) hydrophobic Si (800 °C), (b) hydrophilic Si (800 °C); Figure S2: (a1–c1) SEM images of Hydrophobic SiO_2_ & Si (before & after HCl treatment) particles at different magnifications and (a2–c2) Average particle size distribution Hydrophobic SiO2 & Si (before & after HCl treatment); Figure S3: Nyquist plots from the electrochemical impedance spectroscopy (EIS) measurements for the all-solid-state half-cell of hydrophobic and hydrophilic silicon content; Figure S4: Charge/discharge cycling performance of composite anode containing 5% hydrophilic silicon and 95% graphite for all-solid-state half-cell analysis.
