# Supplementary material for: Development of Porous Silicon(Si) Anode Through Magnesiothermic Reduction of Mesoporous Silica(SiO2) Aerogel for All-Solid-State Lithium-Ion Batteries
Source: Gels. 2025 Apr 21;11(4):304. doi: 10.3390/gels11040304 (PMC12027081; doi:10.3390/gels11040304)
Supplement: Supplementary file 1 [file gels-11-00304-s001.zip › gels-3579510-supplementary.pdf]

Supplementary Information

Development of Porous Silicon(Si) Anode Through Magnesiothermic Reduction of Mesoporous Silica(SiO_2) Aerogel for All-Solid-State Lithium-Ion Batteries

Pratik S. Kapadnis [1], Kangsanin Kim [1], Kisun Nam [1], Yongseon Kim [1], Hyung-Ho Park [2] and Haejin Hwang [1,*]

[1] Department of Materials Science and Engineering, Inha University, Incheon 22212, Republic of Korea; pratiknanoworld@gmail.com (P.S.K.); kks6625@gmail.com (K.K.); skarltjs2@naver.com (K.N.); ys.kim@inha.ac.kr (Y.K.)

[2] Department of Materials Science and Engineering, Yonsei University, Seoul 03722, Republic of Korea; hhpark@yonsei.ac.kr

* Correspondence: hjhwang@inha.ac.kr; Tel.: +82-32-860-7521

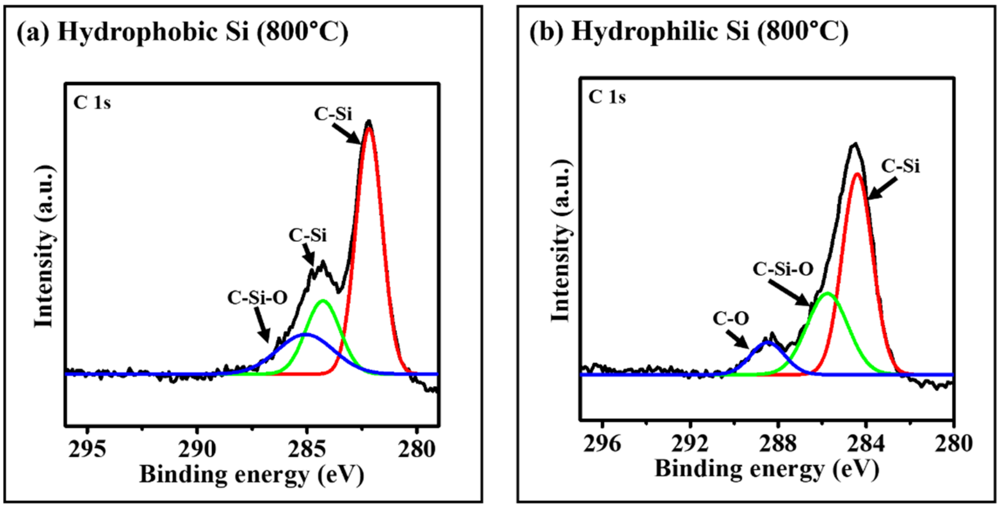

Figure S1. XPS spectrum of C1 S from reduced Si (a) hydrophobic Si (800°C), (b) hydrophilic Si (800°C).

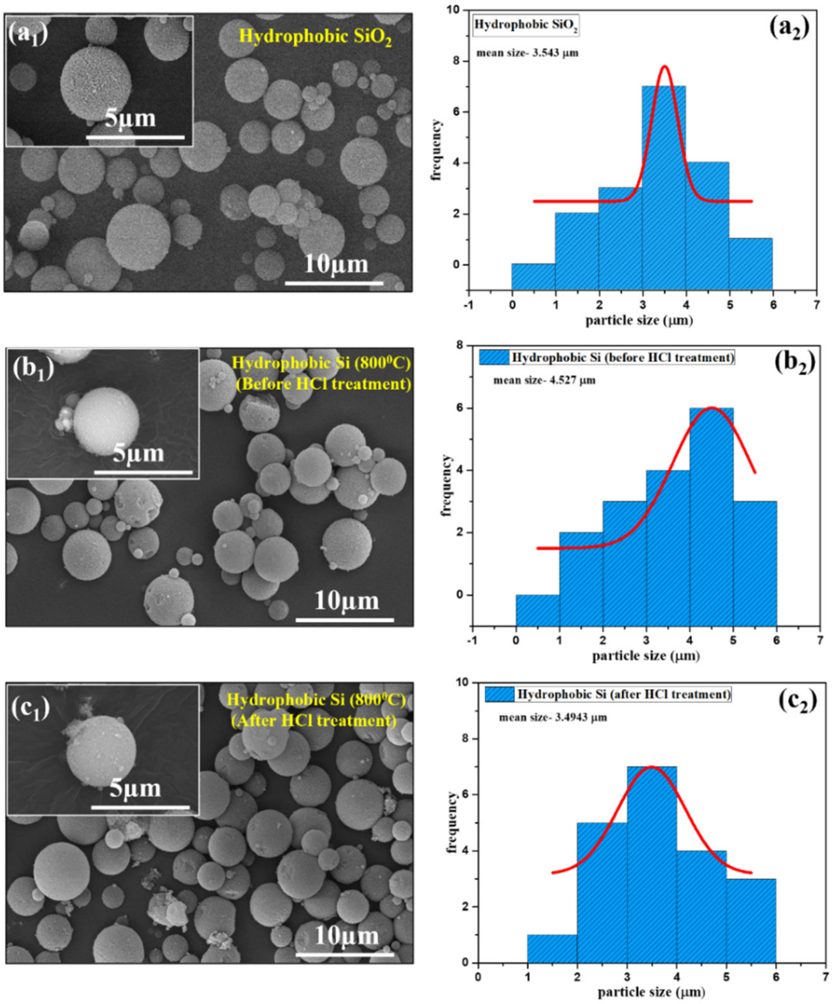

Figure S2. (a_1-c_1) SEM images of Hydrophobic SiO_2 & Si (before & after HCl treatment) particles at different magnifications and (a_2-c_2) Average particle size distribution Hydrophobic SiO_2 & Si (before & after HCl treatment).

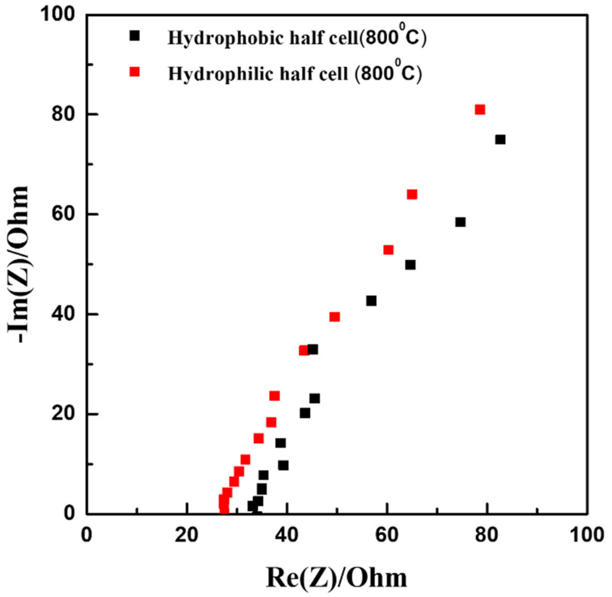

Figure S3. Nyquist plots from the electrochemical impedance spectroscopy (EIS) measurements for the all-solid-state half-cell of hydrophobic and hydrophilic silicon content.

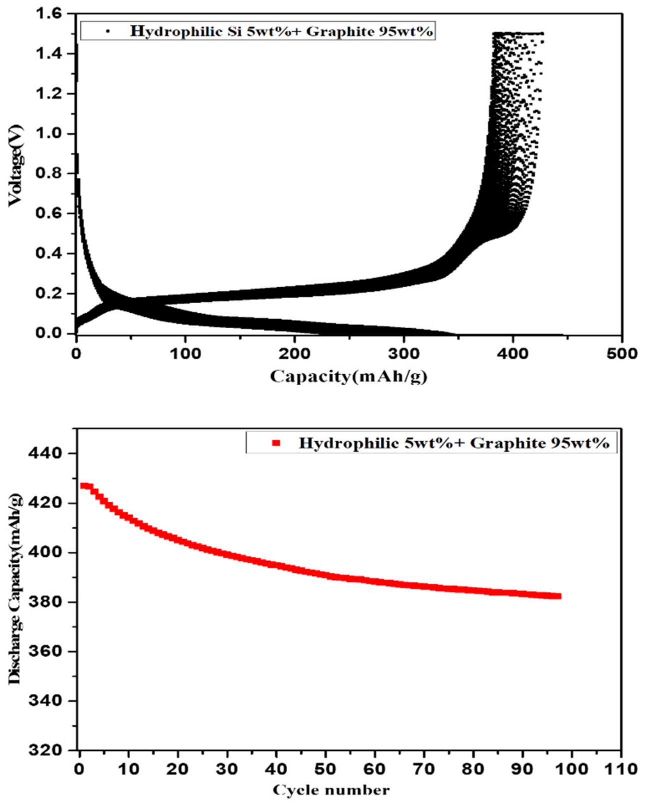

Figure S4. Charge/discharge cycling performance of composite anode containing 5% hydrophilic silicon and 95% graphite for all-solid-state half-cell analysis.
